# Rethinking midwifery education in the Grand Duchy of Luxembourg: Charting a new milestone

**DOI:** 10.18332/ejm/195630

**Published:** 2024-11-26

**Authors:** Joeri Vermeulen, Nicole Weber, Yolande Klein, Kristel Von Laufenberg, Marie Friedel, Ali Ghanchi

**Affiliations:** 1 Department of Life sciences and Medicine, Faculty of Science, Technology and Medicine, University of Luxembourg, Eschsur-Alzette, Grand Duchy of Luxembourg; 2 Department of Public Health, Biostatistics and Medical Informatics Research group, Vrije Universiteit Brussel, Brussels, Belgium; 3 Ecole Nationale de Sante du Luxembourg, Strassen, Grand Duchy of Luxembourg; 4 Association Luxembourgeoise des Sages-Femmes, Luxembourg-ville, Grand Duchy of Luxembourg; 5 Centre Hospitalier du Nord, Ettelbruck, Grand Duchy of Luxembourg; 6 Hiewanspraxis A gudden Hann, Wiltz, Grand Duchy of Luxembourg; 7 Hopitaux Robert Schuman, Kirchberg, Grand Duchy of Luxembourg; 8 Hiewannepraxis am Suden, Esch-sur-Alzette, Grand Duchy of Luxembourg; 9 Institut de recherche Sante et Societe, Universite Catholique de Louvain, Belgium

**Keywords:** midwifery, midwifery education, midwifery students, University of Luxembourg

The Grand Duchy of Luxembourg (GDL) is a landlocked country situated in Western Europe, bordering Belgium, Germany, and France^1^ with a population of 645.397 as of 2022^2^. The country operates under a mandatory social health insurance system that substantially covers medical and maternity expenses^3^. The Luxembourgish health system faces unique challenges among European Union (EU) member states, as it has until recently lacked the capacity to train health personnel and is experiencing shortages in some specialty care areas^1^. Before the founding of the University of Luxembourg in 2003, the GDL lacked a national university, leading its youth to pursue tertiary education abroad. Without a long-standing national university tradition and drawing on the rich experiences of colleagues from various international university systems, the University of Luxembourg has fostered a unique and cohesive institutional culture^4^. Today, the GDL boasts the highest proportion of workers with tertiary qualifications and internationally mobile students, reflecting its investment in higher education capacity-building. This transformation underscores Luxembourg’s commitment to European norms and its unique hyper-diverse, multilingual culture^5^. Nevertheless, Luxembourg depends almost 70% on cross-border healthcare workers, adding to the country’s capacity constraints^6^.

In the last 10 years, the ratio of doctors per 1000 population in the GDL has increased from 2.8 to 3.0 doctors, but it remains below the EU average (4.1)^7^. These ratios are calculated based on the number of residents in the GDL. However, cross-border workers, who make up 46% of employees in Luxembourg, and their families are also covered by the Luxembourg social security system and constitute a significant portion of healthcare beneficiaries in the GDL. As a result, medical coverage and access to care might be considerably lower than the official figures suggest. For 20 years, Luxembourg has offered only the first academic year in Medicine^8^. This may also be related to the low density of the medical workforce^2^. With the creation of new education and training programs, the government aims to attract more of its citizens into the medical, nursing, and midwifery professions thereby increasing the domestic supply of healthcare personnel. In 2020, the GDL initiated a new three-year Bachelor of Medicine, after which graduates pursue postgraduate medical education and training abroad^9^. Postgraduate specialty medical training in Luxembourg is currently limited to general practice, neurology, and oncology. General practice is offered as a four-year program (8 semesters), while neurology and oncology are each offered as five-year programs (10 semesters)^10^. In 2023, the University launched four Bachelor of Science in Nursing programs in the specialized fields of surgery, anesthesia and resuscitation, pediatrics, and mental health. A fifth program, Bachelor of Nursing in General Care, in 3 years, was launched in September 2024^2^, and a Bachelor in Midwifery program will be launched in 2025. In this context, exploring the evolution of midwifery education in Luxembourg becomes imperative, as it directly impacts the quality of care provided to expectant mothers and newborns. To date, the state of midwifery and the revaluation of midwifery education in the GDL has received scant attention in the literature. Insights into how the midwifery profession has evolved over time and the process by which changes to midwifery education have been implemented, maybe useful to other countries that are due to undergo similar reforms^11^.

The aim of this article therefore is to describe the midwifery profession in Luxembourg, identify its challenges and prospects, and highlight the anticipated changes in midwifery education as of 2025, through an analysis of relevant policy and academic texts. This analysis is underpinned by the framework established by Nagórska^12,13^ in the *Medical Professions in International Perspective* series, describing the history, education systems, and principles of practicing the nursing and midwifery professions internationally. The framework consists of the following components: 1) a brief history of midwifery, 2) the system of midwifery education, 3) the legal status of midwifery, 4) midwifery in numbers, and 5) challenges and prospects for midwifery.

## Brief history of midwifery

The first state school for midwives, along with a maternity ward, was established under the auspices of the Medical Council in Pfaffenthal, a suburb of the City of Luxembourg, in 1877. This was followed by the establishment of the *Association Luxembourgeoise des Sages-Femmes* (ALSF) to defend the interests of the profession in 1919. The association brings together midwives from Luxembourg with diverse cultural backgrounds. In 2023, it represented 137 midwives of which 112 were professionally active. Membership is not mandatory and is not linked to the midwives’ license to practice. The association participates in various consultative bodies^14^ for maternity care, such as the Committee for Perinatal Health Monitoring in Luxembourg^15^. Additionally, the ALSF represents Luxemburgish midwives in the International Confederation of Midwives (ICM) and the European Midwives Association. Alongside midwifery educators, the association advises on the curriculum, provides guidance on training needs, and assists with adapting training to the Luxembourgish context. The ALSF adopted the ICM International Code of Ethics for midwives^16^, which addresses the midwife’s ethical mandate to promote the health and well-being of women and newborns within their families and communities^17^. This moral guide for midwives includes details on professional relationships, midwifery practice, upholding professional responsibilities and duties, and how to assure professional integrity of the profession of midwifery^18^.

In the GDL, the state, the medical community, and society recognize the art of midwifery. In certain places, public spaces are named after midwives to perpetuate the memory of individuals and their knowledge in service to successive generations. Since 2019, *D’Hiewanskonscht* (midwifery) has been registered in the national inventory of the intangible cultural heritage of the GDL. Additionally, in 2023, Luxembourg successfully secured the inscription of midwifery on the World Heritage List of the Intangible Cultural Heritage of Humanity of UNESCO^19^.

## System of education for midwives

Midwifery education in the GDL is currently organized at a higher education institution, the *Lycée Technique pour Professions de Santé* (LTPS). The midwifery education is a 3-year direct-entry program of 180 European Credit Transfer and Accumulation System (ECTS) points leading to a *Brevet de Technicien Supérieur* diploma, an advanced technical certificate. The number of admissions into the program is predetermined each year (18 places/year) and the selection process is based on application documents followed by an interview. Previous accreditation commissions in 2017 and 2022 suggested the implementation of a fourth year of study, as the curriculum is very intense for a 3-year programme^20^. This is especially important since new competencies which are more complex and aligned with the competencies described by the ICM were assigned to Luxembourg midwives in 2019^20,21^. The most significant changes include the increased involvement of midwives in prenatal care, as well as an expanded list of medications and tests that they are authorized to prescribe^22^. As a result of these new responsibilities, autonomy has increased allowing midwives to adhere more closely to international standards for maternity care^20^.

In the last decade, midwifery education in most European countries has moved into higher education institutions^23^. The Bologna Declaration and EU Directive 2005/36/EC^24^ were instrumental in bringing about this change. The GDL will comply with this process in the near future^25^. Indications suggest that the degree of implementation of the Directives concerning midwifery education varies across individual EU countries^26^ and within the World Health Organization (WHO) European Region – straddling 53 Member States across Europe and Central Asia – due to differences in healthcare systems, cultural contexts, and resource available^23^. A study on the education level at which midwives complete their education in EU countries reported that in most countries, midwifery training concluded with a Bachelor’s or Master’s degree. In four countries, including Luxembourg, midwives graduated with a diploma^18^. The other three countries, Croatia, Estonia, and Germany, have since moved their midwifery education to the Bachelor’s level. Today, the GDL is the only country in the WHO European Region that does not educate midwives exclusively at the Bachelor’s or Master’s level^27^. Although the current program delivered by the LTPS is in line with Directive 2013/55/EU^28^, the Lair-Hillion^6^ report, which provides a comprehensive overview of the medical and health professions in Luxembourg, recommended upgrading midwifery education to an academic level in order to address both the quantitative and qualitative needs of the healthcare workforce^6^. In all countries, except the GDL, midwives have the opportunity to pursue postgraduate education, such as a Master’s or Doctorate in midwifery or related disciplines. A growth in midwifery research has been observed across Europe^29^, with many midwives in various countries engaging in research and advancing their studies through postgraduate degrees^18^.

As of September 2025, due to major reforms of many other health professions in the GDL, midwifery training will move up to university level. To obtain a Bachelor’s degree, candidates must take part in a 4-year training program of 240 ECTS credits. This decision is aligned with the recommendation of the WHO^30^ and was welcomed by the ALSF because a university-recognized degree will improve the attractivity of midwifery education and allow for greater professional autonomy. While the program at the LTPS is already operating at a higher education level that includes research methodology courses and a final thesis, there is a desire to deepen and expand courses, which is not possible in the format of the current 3-year program. Consequently, it is for this reason and also in line with the 2022 National Accreditation Commission’s recommendation that a fourth year has been requested^20^. Ensuring that training concludes with at least a university Bachelor’s degree is essential in enabling midwives to pursue Master’s or Doctoral studies, either domestically or internationally^31^. This aligns with Luxembourgers’ familiarity with seeking higher education opportunities abroad. Midwifery education in the GDL will be fully academicized by 2029 and will be offered exclusively at the University of Luxembourg.

As in line with international requirements^23^, the University of Luxembourg’s midwifery curriculum will be meticulously designed based on the latest evidence and learning will be facilitated through simulation exercises. This is paired with practical training alongside seasoned midwives across various practice settings, ranging from home to hospital births. Under close mentorship and guidance, students will develop the skills needed to become qualified midwives. In most countries, research and evidence-based practice is part of midwifery education^23^. With a strong focus on evidence-based practice, these programs are designed to equip midwives to meet the demands of modern maternity care. Consequently, the midwifery course of the University of Luxembourg will be no different, focusing on family-centered care and evidence based midwifery. A particular emphasis will be given to interprofessional collaboration and the provision of safe and high-quality perinatal care. The well-being and quality of life of students will also be taken into consideration.

## Legal status of midwifery

Midwifery practice in the GDL is determined by the European Directives, outlining midwifery’s particular competencies, which are specified in national legislation. Specifically, in the Grand-Ducal Regulation of 22 November 2019 and the Law of 29 June 2023 determining the status, duties, and rules governing the practice of the midwifery profession, it is stated that midwives in the GDL are autonomous and competent to practice independently in an uncomplicated pregnancy, labor and childbirth^22,32^. The midwifery profession in the GDL is a regulated profession that includes diagnostic and prescriptive authority. Midwives primarily practice in hospitals but also offer services privately or in family planning centers. They specialize in normal pregnancies and are involved in all stages of childbirth, from preparation to postnatal home care^20,22^. In the GDL, midwives are specialists in eutocia and provide care in both hospital and primary care settings. They offer counseling and supervision to healthy women and newborns throughout the preconception, prenatal, intrapartum, and postnatal periods. However, the development of pre- and postnatal care within primary healthcare settings remains a challenge, as hospitals remain the primary employers of midwives^6^.

A recent study, carried out in 2019 across Europe, highlighted significant under-utilization of midwives, whose professional duties include monitoring normal pregnancies and performing normal births, including episiotomies^18^. In the GDL, midwives are not consistently fulfilling these responsibilities across all maternity units, as Luxembourgish pregnant women predominantly receive care from an obstetrician^6^. The choice of having an obstetrician as the primary care provider affects the place of birth, as pregnant women usually give birth in the clinic with which the obstetrician is affiliated^33^. To qualify for a prenatal allowance from the government, women are required to attend five prenatal visits with an obstetrician. During pregnancy, 99.6% of women are monitored by a healthcare professional, and the majority (92.6%) of them consult a healthcare professional during the first trimester of pregnancy^15^. Moreover, a medical prescription for midwifery care is only required for monitoring pathological situations. For uncomplicated situations, midwifery care is reimbursed directly by the Luxembourg social security^33,34^.

## Midwifery in numbers

In 2019, there were 234 midwives actively practicing in the GDL, all female, and 37.1% of them worked fulltime. Amongst professionally active midwives, 38% were Luxembourgish, while France and Belgium together contributed more than 56% of the midwives in the GDL^6^. The ratio of midwives in the GDL has remained unchanged between 2010 and 2020 at 0.8 midwives per 100000 inhabitants^35^. By contrast, the number of midwifery graduates per 100000 inhabitants in 2018 was amongst the lowest in the EU (less than 1.0 in Germany, the Netherlands, Austria, Luxembourg, Italy, Spain, and Cyprus)^35^.

Nevertheless, the recruitment pool, which covers the entire territory of the GDL, has a high demand for midwives today and will have in the coming years. Between 2019 and 2034, it is projected that there will be 81 anticipated departures out of a total of 234 midwives, i.e. 34.6% of the current workforce^20^. To replace these departures, the GDL will need to produce an average of 5 certified midwives per year, exceeding the number of graduates in recent years. Furthermore, the number of students is slightly decreasing, while demand rises. Reconsidering the training program is therefore fully justified because current recruitment is barely meeting demand^20^. A university-affiliated midwifery degree course may encourage more students to apply.

In 2019, 7108 births were recorded at the four maternity hospitals in the GDL^33^. The size of the maternity hospitals varies significantly, ranging from 770 births per year to 3019 births in 2019^15^. One of the typical characteristics of maternity care in the GDL is its large proportion of foreign women who give birth in the country. In 2019, 23.5% of pregnant women were of non-European Union nationality. Among the women from the European Union giving birth in the GDL, the majority had either Portuguese, French or Belgian nationality. Another characteristic of Luxembourg is the proportion of non-resident women who receive medical care as numerous cross-border workers commute daily for work. Consequently, they are insured by the National Health Fund and have the option to receive medical treatment either in their country of residence or in Luxembourg. Between 2017 and 2019, cross-border women giving birth in the GDL accounted for 14.0% of all births^15^.

The number of planned home births was 6 in 2019. Meanwhile, between 2017 and 2019, 21 unplanned home births occurred^15^. In the latest perinatal epidemiology report, covering data from 2014–2016, the average cesarean section rate was 32%, and the rate of instrumental vaginal births was 11.4% in the GDL^15^. There has been a significant decrease in episiotomy rates over the last decade. In 2019, 13.7% of vaginal births involved an episiotomy, and this rate dropped to 10.8% when stand-alone episiotomies, excluding associated tears are considered^15^.

**Table 1:** Graduate midwives, 2010 and 2020

*Country*	*Total number*		*Ratio per 100000 inhabitants*	
	*2010*	*2020*	*2010*	*2020*
Belgium	648	648	5.9	5.6
Bulgaria	99	199	1.3	2.9
Czechia	229	195	2.2	1.8
Denmark^a^	162	165	2.9	2.8
Germany	596	750	0.7	0.9
Estonia	31	30	2.3	2.3
Ireland	186	128	4.1	2.6
Greece^a^	236	235	2.1	2.2
Spain	353	360	0.8	0.8
France	877	898	1.4	1.3
Croatia^b^	190	82	4.4	2.0
Italy	804	456	1.4	0.8
Cyprus^a^	0	0	-	-
Latvia	13	42	0.6	2.2
Lithuania	23	33	0.7	1.2
Luxembourg	4	5	0.8	0.8
Hungary	52	229	0.5	2.4
Malta	19	11	4.6	2.1
Netherlands^b^	141	150	0.9	0.9
Austria	91	74	1.1	0.8
Poland^b^	1757	1471	4.6	3.9
Portugal^c^				
Romania	155		0.8	
Slovenia^b^	26	37	1.3	1.8
Slovakia	82	59	1.5	1.1
Finland	177	196	3.3	3.5
Sweden	279	345	3.0	3.3
Iceland^a^	10	10	3.1	2.8
Liechtenstein	0	0	-	-
Norway	89	145	1.8	2.7
Switzerland	68	203	0.9	2.4
Montenegro^c^				
North Macedonia	14	24	0.7	1.2
Serbia	382	364	5.3	5.3
Turkey	1262	3479	1.7	4.2

a 2019 instead of 2020.

b 2018 instead of 2020.

c No data available.

Source: Eurostat. Healthcare personnel statistics - nursing and caring professionals, 2022.

## Challenges and prospects

This article aimed to assess the current state of midwifery in the GDL and use these insights to develop a new educational program that meets the future needs of the profession. Ensuring that learning outcomes are relevant and sustainable will enable Luxembourg to address existing challenges and adequately prepare its midwifery workforce.

The Luxembourg government initiated reforms in 2021 to enhance the attractiveness of healthcare professions, particularly focusing on nurses and midwives. These reforms aimed to address current and anticipated workforce shortages and improve healthcare delivery^36^. In 2023, WHO/Europe ministers from the smallest member states, including Luxembourg, committed to improving health outcomes through greater investment in healthcare worker education and retention^37^. Strengthening recruitment and retention in midwifery is key to expanding access to safe, respectful maternity care^11^. To attract candidates to the midwifery profession, it is essential to effectively promote its competencies, roles, and opportunities^38^. However, Luxembourg faces significant recruitment challenges, notably the difficulty of attracting non-Luxembourgish students due to the bilingual language requirements (French and German). Additionally, the existing midwifery program is recognized only as a two-year degree, making salaries less competitive^20^. Consequently, 50% of midwives working in Luxembourg are from neighboring countries^20^. With WHO targeting a 50% reduction in foreign recruitment by 2030, proactive policies are needed to encourage local students to pursue midwifery and enhance the program’s value to improve the profession’s image^6^.

A key solution lies in elevating midwifery education to a university degree^39^. In recent years, many countries have transitioned midwifery education to universities to ensure autonomy, professionalization, and readiness for future challenges^25^. A university degree could not only enhance the profession’s attractiveness to graduates but also better prepare midwives for delivering safe, evidence-based care^40^. In Luxembourg, the shift to a university model can reinforce professional identity and offer continuous updates on best practices, thus driving transformative change in the sector. The recent academization of midwifery posed significant challenges for Germany; universities needed to innovate in curricular and practical teaching, fill professorships, and secure adequate resources^41^. The anticipated changes will have far-reaching effects on midwifery care quality in Luxembourg. By elevating midwifery education to a university level, midwives will be better equipped to meet the complex demands of modern maternal healthcare, ensuring that they are able to deliver high-quality, evidence-based care^42^. This approach may help to address current recruitment challenges by attracting more students and improving retention rates. Additionally, the combination of extended clinical placements and an emphasis on interprofessional education will ensure that midwives are not only clinically competent but also confident in collaborating across healthcare settings, enhancing the overall quality of care provided to women and families^42^.

The University of Luxembourg is currently developing a four-year Bachelor’s degree in midwifery, which aims to provide students with the necessary knowledge, skills, and behaviors for safe and competent practice. High-quality clinical experience is one of the most critical factors in midwifery education, as it is essential for developing the ability to provide competent care^43^. The program will incorporate extended clinical placements to ensure students gain hands-on experience in all aspects of midwifery care, from pregnancy to postpartum. Collaboration between the University of Luxembourg and local healthcare institutions – such as hospitals, clinics, and community birth settings – through structured placements, mentorship, and interprofessional learning, will ensure that students gain practical experience in real-life environments. This will build their competence, confidence, professional identity, and teamwork skills, enabling them to deliver competent, evidence-based care^43^.

The emphasis on interprofessional education and simulation platforms will further enhance learning and clinical competence, aligning with international standards. The objectives of the midwifery 4-year Bachelor’s degree at the University of Luxembourg is to provide students with the knowledge, skills, and attitudes of future midwives so that they are able to practice all aspects of the midwifery profession safely and competently as defined by law in Luxembourg. The ultimate aim is to empower students so that they will be able to deliver healthcare to a high standard to all women of childbearing age and their families. The program will be designed to encourage critical thinking in an academic setting and form solid foundations for students to develop a sound professional practice that best prepares them to face everyday real-world challenges of the midwifery profession and for future study. For long-term success, continuous review and adaptation of the midwifery curriculum are essential. Collaboration between educators, healthcare providers, and stakeholders will ensure the curriculum meets evolving health challenges and remains aligned with European and national qualification frameworks. Stakeholder engagement will also help bridge the gap between theory and practice, ensuring that midwifery education remains responsive to societal needs^44,45^. While the positive impact of quality midwifery education on maternal and newborn health has been acknowledged by the WHO, it highlights the need to engage with stakeholders in education^46^. The clear symbiosis of quality midwifery education with quality care for women and their families cannot be ignored^47^. A continuing dialogue with organizational management in the clinical area is important for establishing joint positions across academic and healthcare institutions.

Our aim is to foster collaboration between practicing midwives and educators to integrate clinical leadership with research, advancing midwife-led continuity services and delivering sustainable, evidence-based care^44^. In line with the theory of participatory curriculum development framework, stakeholder engagement will be promoted. The provision and maintenance of quality education and practice require shared responsibility between education providers and healthcare services^48^. Professional programs benefit from this involvement by maintaining a strong connection between theory and practice. Inclusive representation creates a curriculum that is responsive to changes within knowledge and societal expectations^49^.

Beyond the Bachelor’s degree, Luxembourg could also consider the possibility of introducing postgraduate programs in midwifery. A Master’s degree can prepare midwives for advanced roles in management, research, education, and clinical specialization^39^. There is a pressing need to design Master’s programs tailored to the field. Additionally, it is crucial to consider specific advanced roles for midwife practitioners and develop corresponding educational programs. A clear pathway towards clinical specialization in midwifery at an advanced level needs to be established, in alignment with health policy regulations. For example, over the past decade, Ireland has established high-quality midwifery education programs that ensure graduate midwives can deliver safe and competent care^45^. This has led to increased opportunities for registered midwives to pursue higher level qualifications, with several universities offering Master’s and Doctoral programs in midwifery. In Ireland, midwives can specialize in various areas such as normal and complicated pregnancy, birth, postpartum care, and reproductive health^44^. Moreover, advanced midwife practitioners are playing a pivotal role in advancing midwifery practice in some settings^45^. Furthermore, there is a need to establish doctoral programs for midwives in fields such as social health sciences, psychology, and education. This would contribute to the growth of midwifery research across Europe. While midwives in many countries have the opportunity to engage in research and pursue postgraduate studies, including Master’s and Doctoral degrees, Luxembourg notably lacks such opportunities.

The experiences of other European countries demonstrate that a strong theoretical foundation at the university level, positively influences the professional identity of midwives^50^. Future research in Luxembourg should explore how midwives perceive their professional identity within this new educational framework. Insights from this study could guide midwifery educators globally in shaping their programs and addressing the challenges midwives face in practice. While the global trend toward university-level midwifery education is clear, it is important to recognize that educational models must be adapted to the specific context of each country. Differences in healthcare systems, regulations, and scope of practice require tailored approaches to ensure that education aligns with local needs^51^.

Currently, midwives in Luxembourg are under-utilized, as many of their competencies, such as monitoring normal pregnancies and issuing medical certificates, are not fully recognized by the authorities or employers^6^. Independent midwives have no access to hospitals to support the women they care for during labor and childbirth. Currently, there are few options for women to access midwives directly and community midwifery care is not widely available. Ongoing legal reforms are addressing these issues, and the introduction of a university-accredited degree is expected to further elevate the recognition of midwives’ competencies by healthcare professionals and administrators.

As the midwifery profession evolves, Luxembourg’s experience may offer valuable lessons for other countries facing similar challenges. The shift toward university-level midwifery education, combined with legal reforms and stakeholder collaboration, has the potential to strengthen the profession both nationally and globally. The identification of midwifery as a distinct profession and the implementation of transformative practices have led to substantial changes in midwifery education in Australia^48^. Knowledge sharing through exchange programs, such as ERASMUS, can also foster international co-operation and raise standards of midwifery education worldwide.

## Conclusion

This article highlighted the urgent need to reform midwifery education in Luxembourg, focusing on recruitment, retention, and elevating midwifery training to a university level. As Luxembourg is the last country in the EU to implement Bachelor’s or Master’s level midwifery education, it has a unique opportunity to learn from the experiences of other countries and consider the obstacles they encounter. Addressing current challenges, such as language barriers, and under-utilization of midwife competencies, will require targeted policies that emphasize professional development and the creation of advanced educational programs. By aligning with international standards and fostering collaboration between educators, healthcare institutions, and stakeholders, Luxembourg can futureproof its midwifery workforce and contribute to the global advancement of midwifery care. The emphasis on collaboration, stakeholder engagement, and the creation of advanced educational pathways offers a blueprint for strengthening midwifery education globally.

## Data Availability

Data sharing is not applicable to this article as no new data were created.
